# Supplementary figures and images for: COVID-19 hospitalisations and all-cause mortality by risk group in Finland
Source: PLoS One. 2023 May 23;18(5):e0286142. doi: 10.1371/journal.pone.0286142 (PMC10204977; doi:10.1371/journal.pone.0286142)

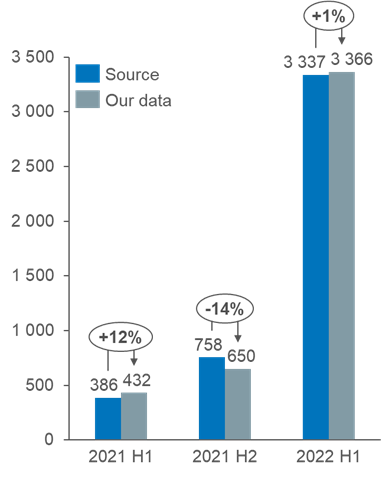

Supplement: S1 Fig — (TIF) [file pone.0286142.s001.tif]
