# Supplementary material for: COVID-19 hospitalisations and all-cause mortality by risk group in Finland
Source: PLoS One. 2023 May 23;18(5):e0286142. doi: 10.1371/journal.pone.0286142 (PMC10204977; doi:10.1371/journal.pone.0286142)
Supplement: S1 Table — (PDF) [file pone.0286142.s002.pdf]

S1 Table. Difference between the number of patients with a COVID-19 infection identified from THL registers (Hilmo and Avohilmo) and patients identified only in the TTR (primary and specialty care separately) (n = THL-TTR, % = 1-(THL-TTR)/THL).

	Primary care patients						Specialty care patients					
**H1 2021**	**18–59**		**60+**		**18+**		**18–59**		**60+**		**18+**	
All	30	72,70 %	188	70,70 %	218	71,00 %	93	92,60 %	72	92,90 %	165	92,70 %
No risk	14	79,40 %	56	61,90 %	70	67,40 %	77	91,80 %	32	91,80 %	109	91,80 %
Min. 1 risk	16	61,90 %	132	73,30 %	148	72,40 %	16	94,80 %	40	93,60 %	56	94,00 %
Cancer	4	42,90 %	33	75,40 %	37	73,80 %	1	96,20 %	10	91,50 %	11	92,40 %
Chronic lung disease	3	70,00 %	37	68,40 %	40	68,50 %	3	96,90 %	12	92,90 %	15	94,30 %
CKD	2	50,00 %	7	85,10 %	9	82,40 %	1	95,20 %	5	92,10 %	6	92,90 %
CV diseases	6	33,30 %	70	76,00 %	76	74,80 %	1	98,50 %	16	94,50 %	17	95,30 %
Diabetes	4	50,00 %	33	76,60 %	37	75,20 %	5	93,60 %	10	94,70 %	15	94,30 %
Hypertension	8	33,30 %	99	72,30 %	107	71,10 %	7	94,50 %	25	93,80 %	32	94,00 %
Neurological disorders or diseas	1	66,70 %	8	68,00 %	9	67,90 %	2	81,80 %	1	92,90 %	3	88,00 %
Organ or stem cell transplant	1	87,50 %	5	80,80 %	6	82,40 %	5	86,10 %	2	95,60 %	7	91,40 %
	**Primary care patients**						**Specialty care patients**					
**H2 2021**	**18–59**		**60+**		**18+**		**18–59**		**60+**		**18+**	
All	44	68,80 %	238	73,50 %	282	72,80 %	127	92,40 %	102	92,30 %	229	92,30 %
No risk	28	70,80 %	51	68,50 %	79	69,40 %	105	91,70 %	45	89,40 %	150	91,10 %
Min. 1 risk	16	64,40 %	187	74,60 %	203	74,00 %	22	94,50 %	57	93,70 %	79	93,90 %
Cancer	5	28,60 %	38	76,30 %	43	74,30 %	3	94,50 %	13	93,50 %	16	93,70 %
Chronic lung disease	3	78,60 %	45	77,60 %	48	77,70 %	5	95,50 %	15	94,40 %	20	94,70 %
CKD	0	100,00 %	19	78,20 %	19	78,70 %	2	92,90 %	12	87,60 %	14	88,80 %
CV diseases	9	52,60 %	113	75,70 %	122	74,80 %	6	93,70 %	35	92,80 %	41	93,00 %
Diabetes	3	70,00 %	66	71,90 %	69	71,80 %	6	94,20 %	22	92,70 %	28	93,10 %
Hypertension	7	65,00 %	134	73,60 %	141	73,20 %	7	95,50 %	42	92,90 %	49	93,40 %
Neurological disorders or diseas	0	100,00 %	11	71,80 %	11	72,50 %	0	100,00 %	4	84,60 %	4	90,50 %
Organ or stem cell transplant	2	33,30 %	10	77,30 %	12	74,50 %	7	85,10 %	4	93,00 %	11	89,40 %
	**Primary care patients**						**Specialty care patients**					
**H1 2022**	**18–59**		**60+**		**18+**		**18–59**		**60+**		**18+**	
All	56	77,40 %	696	81,30 %	751	81,10 %	580	79,80 %	629	89,30 %	1 208	86,20 %
No risk	26	77,00 %	175	72,00 %	201	72,80 %	440	75,70 %	343	74,50 %	782	75,20 %
Min. 1 risk	30	77,80 %	521	83,20 %	550	83,00 %	140	86,70 %	286	93,70 %	425	92,40 %
Cancer	3	85,70 %	140	81,80 %	143	81,90 %	30	85,80 %	102	92,00 %	131	91,20 %
Chronic lung disease	6	86,40 %	118	84,20 %	123	84,40 %	40	86,70 %	62	94,70 %	102	93,10 %
CKD	1	88,90 %	70	78,80 %	70	79,30 %	20	83,20 %	37	93,10 %	57	91,30 %
CV diseases	4	90,00 %	330	83,00 %	333	83,20 %	37	87,50 %	156	94,20 %	193	93,50 %
Diabetes	10	72,20 %	164	83,00 %	174	82,60 %	32	89,20 %	91	93,90 %	123	93,10 %
Hypertension	21	68,70 %	382	82,60 %	403	82,20 %	55	86,40 %	195	93,60 %	249	92,80 %
Neurological disorders or diseas	0	100,00 %	20	87,40 %	20	88,00 %	3	92,90 %	13	93,30 %	16	93,20 %
Organ or stem cell transplant	2	83,30 %	33	82,60 %	34	83,10 %	31	85,00 %	40	90,60 %	70	88,90 %

CV = cardiovascular, CKD = chronic kidney disease, THL = The National Institute for Health and Welfare, TTR = Finnish National Infectious Diseases Register
