# Supplementary material for: COVID-19 hospitalisations and all-cause mortality by risk group in Finland
Source: PLoS One. 2023 May 23;18(5):e0286142. doi: 10.1371/journal.pone.0286142 (PMC10204977; doi:10.1371/journal.pone.0286142)
Supplement: S2 Table — (PDF) [file pone.0286142.s003.pdf]

S2 Table. Difference between days in hospital care for patients identified from THL registers (Hilmo and Avohilmo) and for patients identified only in the TTR (primary and specialty care separately) (n = THL-TTR, % = 1-(THL-TTR)/THL).

	Primary care days in hospital						Specialty care days in hospital					
**H1 2021**	**18–59**		**60+**		**18+**		**18–59**		**60+**		**18+**	
All	663	56,50 %	6288	53,60 %	6951	53,80 %	1168	89,20 %	1872	84,40 %	3040	86,70 %
No risk	222	72,20 %	1263	48,70 %	1485	54,50 %	740	90,50 %	565	87,30 %	1305	89,40 %
Min. 1 risk	441	39,10 %	5025	54,60 %	5466	53,70 %	428	86,00 %	1307	82,60 %	1735	83,60 %
Cancer	156	16,60 %	1606	54,30 %	1762	52,40 %	25	91,10 %	257	81,60 %	282	83,20 %
Chronic lung disease	68	51,80 %	1342	53,00 %	1410	53,00 %	55	94,00 %	225	87,50 %	280	89,70 %
CKD	22	60,00 %	281	69,60 %	303	69,00 %	7	97,10 %	228	71,60 %	235	77,50 %
CV diseases	210	15,30 %	2838	59,40 %	3048	57,90 %	197	77,60 %	767	80,70 %	964	80,20 %
Diabetes	53	47,00 %	1460	57,20 %	1513	56,90 %	288	68,00 %	497	80,70 %	785	77,40 %
Hypertension	191	18,70 %	3950	53,30 %	4141	52,30 %	231	84,20 %	878	82,00 %	1109	82,50 %
Neurological disorders or diseas	27	27,00 %	263	54,10 %	290	52,50 %	40	69,20 %	73	71,40 %	113	70,60 %
Organ or stem cell transplant	37	73,90 %	256	51,90 %	293	56,50 %	114	76,10 %	178	75,10 %	292	75,50 %
	**Primary care days in hospital**						**Specialty care days in hospital**					
**H2 2021**	**18–59**		**60+**		**18+**		**18–59**		**60+**		**18+**	
All	866	37,20 %	9411	49,90 %	10277	49,00 %	2595	81,60 %	3483	77,40 %	6078	79,40 %
No risk	377	43,80 %	1526	52,70 %	1903	51,20 %	1585	84,00 %	1266	75,70 %	2851	81,20 %
Min. 1 risk	489	30,90 %	7885	49,30 %	8374	48,50 %	1010	75,80 %	2217	78,30 %	3227	77,60 %
Cancer	37	9,80 %	1547	55,40 %	1584	54,90 %	268	65,50 %	703	70,80 %	971	69,50 %
Chronic lung disease	97	34,90 %	1832	53,00 %	1929	52,30 %	110	88,50 %	530	79,90 %	640	82,20 %
CKD	0	100,00 %	869	53,60 %	869	53,90 %	84	77,40 %	384	69,80 %	468	71,50 %
CV diseases	271	18,10 %	4864	49,50 %	5135	48,50 %	255	77,30 %	1354	75,90 %	1609	76,10 %
Diabetes	126	40,60 %	2924	44,70 %	3050	44,60 %	192	80,90 %	747	79,50 %	939	79,80 %
Hypertension	301	33,40 %	5927	46,50 %	6228	46,00 %	485	75,00 %	1531	77,70 %	2016	77,10 %
Neurological disorders or diseas	0	100,00 %	574	45,50 %	574	45,90 %	0	100,00 %	308	34,70 %	308	48,10 %
Organ or stem cell transplant	68	10,50 %	407	52,80 %	475	49,40 %	324	60,20 %	275	71,10 %	599	66,00 %
	**Primary care days in hospital**						**Specialty care days in hospital**					
**H1 2022**	**18–59**		**60+**		**18+**		**18–59**		**60+**		**18+**	
All	1425	52,00 %	25241	62,30 %	26666	61,80 %	5326	71,90 %	9832	79,00 %	15 158	77,00 %
No risk	415	61,90 %	3835	60,90 %	4250	61,00 %	2937	71,70 %	3653	65,90 %	6590	68,80 %
Min. 1 risk	1010	46,20 %	21406	62,50 %	22416	62,00 %	2389	72,10 %	6179	82,90 %	8568	80,90 %
Cancer	68	71,40 %	5742	59,80 %	5810	59,90 %	664	68,40 %	2110	80,30 %	2774	78,30 %
Chronic lung disease	136	60,70 %	4577	64,10 %	4713	64,00 %	510	73,90 %	1365	85,00 %	1875	83,00 %
CKD	52	45,30 %	2495	59,00 %	2547	58,80 %	295	75,70 %	836	82,40 %	1131	81,00 %
CV diseases	167	68,50 %	13777	62,10 %	13944	62,20 %	957	66,40 %	3352	84,10 %	4309	82,00 %
Diabetes	269	47,60 %	6004	64,80 %	6273	64,30 %	506	79,50 %	1767	85,30 %	2273	84,30 %
Hypertension	732	39,90 %	16039	61,00 %	16771	60,40 %	763	79,00 %	4294	82,40 %	5057	82,00 %
Neurological disorders or diseas	0	100,00 %	802	69,80 %	802	70,30 %	57	78,20 %	271	81,30 %	328	80,80 %
Organ or stem cell transplant	59	45,90 %	1171	64,40 %	1230	63,80 %	715	65,50 %	771	81,50 %	1486	76,20 %
