# Supplementary material for: COVID-19 hospitalisations and all-cause mortality by risk group in Finland
Source: PLoS One. 2023 May 23;18(5):e0286142. doi: 10.1371/journal.pone.0286142 (PMC10204977; doi:10.1371/journal.pone.0286142)
Supplement: S3 Table — (PDF) [file pone.0286142.s004.pdf]

S3 Table. Risk groups and identification of risk groups from data.

	ICD-10 code	Finnish Classification of Surgical Procedures
Diabetes	E10*-E11*	
CV diseases	I20*-I28*, I42*, I50*, I61*, I63*	
Hypertension	I10*	
Chronic lung disease	J40*-J47*	
Organ or stem cell transplant		WW*, FQ*, JJC*, KAS*, GDG_10, GDG_13, GDG_96
Cancer diagnosis (with a health care visit in 2020-2021)	C00*-C97*	
CKD	N18*-N19*	
Neurological disorders or diseases	G20*, G35*, G80*, Q90*	

CV = cardiovascular, CKD = chronic kidney disease.
