# Supplementary material for: COVID-19 hospitalisations and all-cause mortality by risk group in Finland
Source: PLoS One. 2023 May 23;18(5):e0286142. doi: 10.1371/journal.pone.0286142 (PMC10204977; doi:10.1371/journal.pone.0286142)
Supplement: S4 Table — All groups are compared to the no risk -group. (PDF) [file pone.0286142.s005.pdf]

**S4 Table. P-values of statistical testing for IHR and CFR. All groups are compared to the no risk -group.**

| | Primary care hospitalised patients | | | Specialty care hospitalised patients | | | Deaths | | |
|---|---|---|---|---|---|---|---|---|---|
| **H1 2021** | **18–59** | **60+** | **18+** | **18–59** | **60+** | **18+** | **18–59** | **60+** | **18+** |
| All | 0.0120 | <0.001 | <0.001 | <0.001 | <0.001 | <0.001 | 0.0418 | <0.001 | <0.001 |
| No risk | 1.000 | 1.000 | 1.000 | 1.000 | 1.000 | 1.000 | 1.000 | 1.000 | 1.000 |
| Min. 1 risk | <0.001 | <0.001 | <0.001 | <0.001 | <0.001 | <0.001 | <0.001 | <0.001 | <0.001 |
| Cancer | <0.001 | <0.001 | <0.001 | <0.001 | <0.001 | <0.001 | <0.001 | <0.001 | <0.001 |
| Chronic lung disease | <0.001 | <0.001 | <0.001 | <0.001 | <0.001 | <0.001 | 0.526 | <0.001 | <0.001 |
| CKD | <0.001 | <0.001 | <0.001 | <0.001 | <0.001 | <0.001 | <0.001 | <0.001 | <0.001 |
| CV diseases | <0.001 | <0.001 | <0.001 | <0.001 | <0.001 | <0.001 | <0.001 | <0.001 | <0.001 |
| Diabetes | <0.001 | <0.001 | <0.001 | <0.001 | <0.001 | <0.001 | <0.001 | <0.001 | <0.001 |
| Hypertension | <0.001 | <0.001 | <0.001 | <0.001 | <0.001 | <0.001 | <0.001 | <0.001 | <0.001 |
| Neurological disorders or diseas | <0.001 | <0.001 | <0.001 | <0.001 | 0.136 | <0.001 | 0.823 | <0.001 | <0.001 |
| Organ or stem cell transplant | <0.001 | <0.001 | <0.001 | <0.001 | <0.001 | <0.001 | 0.070 | <0.001 | <0.001 |
| | **Primary care hospitalised patients** | | | **Specialty care hospitalised patients** | | | **Deaths** | | |
| **H2 2021** | **18–59** | **60+** | **18+** | **18–59** | **60+** | **18+** | **18–59** | **60+** | **18+** |
| All | 0.030 | <0.001 | <0.001 | <0.001 | <0.001 | <0.001 | 0.030 | <0.001 | <0.001 |
| No risk | 1.000 | 1.000 | 1.000 | 1.000 | 1.000 | 1.000 | 1.000 | 1.000 | 1.000 |
| Min. 1 risk | <0.001 | <0.001 | <0.001 | <0.001 | <0.001 | <0.001 | <0.001 | <0.001 | <0.001 |
| Cancer | <0.001 | <0.001 | <0.001 | <0.001 | <0.001 | <0.001 | <0.001 | <0.001 | <0.001 |
| Chronic lung disease | <0.001 | <0.001 | <0.001 | <0.001 | <0.001 | <0.001 | <0.001 | <0.001 | <0.001 |
| CKD | <0.001 | <0.001 | <0.001 | <0.001 | <0.001 | <0.001 | <0.001 | <0.001 | <0.001 |
| CV diseases | <0.001 | <0.001 | <0.001 | <0.001 | <0.001 | <0.001 | <0.001 | <0.001 | <0.001 |
| Diabetes | <0.001 | <0.001 | <0.001 | <0.001 | <0.001 | <0.001 | <0.001 | <0.001 | <0.001 |
| Hypertension | <0.001 | <0.001 | <0.001 | <0.001 | <0.001 | <0.001 | <0.001 | <0.001 | <0.001 |
| Neurological disorders or diseas | 0.3048 | <0.001 | <0.001 | <0.001 | <0.001 | <0.001 | <0.001 | <0.001 | <0.001 |
| Organ or stem cell transplant | 0.084 | <0.001 | <0.001 | <0.001 | <0.001 | <0.001 | <0.001 | <0.001 | <0.001 |
| | **Primary care hospitalised patients** | | | **Specialty care hospitalised patients** | | | **Deaths** | | |
| **H1 2022** | **18–59** | **60+** | **18+** | **18–59** | **60+** | **18+** | **18–59** | **60+** | **18+** |
| All | <0.001 | <0.001 | <0.001 | <0.001 | <0.001 | <0.001 | <0.001 | <0.001 | <0.001 |
| No risk | 1.000 | 1.000 | 1.000 | 1.000 | 1.000 | 1.000 | 1.000 | 1.000 | 1.000 |
| Min. 1 risk | <0.001 | <0.001 | <0.001 | <0.001 | <0.001 | <0.001 | <0.001 | <0.001 | <0.001 |
| Cancer | <0.001 | <0.001 | <0.001 | <0.001 | <0.001 | <0.001 | <0.001 | <0.001 | <0.001 |
| Chronic lung disease | <0.001 | <0.001 | <0.001 | <0.001 | <0.001 | <0.001 | <0.001 | <0.001 | <0.001 |
| CKD | <0.001 | <0.001 | <0.001 | <0.001 | <0.001 | <0.001 | <0.001 | <0.001 | <0.001 |
| CV diseases | <0.001 | <0.001 | <0.001 | <0.001 | <0.001 | <0.001 | <0.001 | <0.001 | <0.001 |
| Diabetes | <0.001 | <0.001 | <0.001 | <0.001 | <0.001 | <0.001 | <0.001 | <0.001 | <0.001 |
| Hypertension | <0.001 | <0.001 | <0.001 | <0.001 | <0.001 | <0.001 | <0.001 | <0.001 | <0.001 |
| Neurological disorders or diseas | <0.001 | <0.001 | <0.001 | <0.001 | <0.001 | <0.001 | <0.001 | <0.001 | <0.001 |
| Organ or stem cell transplant | <0.001 | <0.001 | <0.001 | <0.001 | <0.001 | <0.001 | <0.001 | <0.001 | <0.001 |

CV = cardiovascular, CKD = chronic kidney disease.
