# Supplementary material for: COVID-19 hospitalisations and all-cause mortality by risk group in Finland
Source: PLoS One. 2023 May 23;18(5):e0286142. doi: 10.1371/journal.pone.0286142 (PMC10204977; doi:10.1371/journal.pone.0286142)
Supplement: S5 Table — (PDF) [file pone.0286142.s006.pdf]

S5 Table. COVID-19 vaccination coverage in Finland (n, % of population).

	Vaccination dose	Time	18+		18–59		60+	
			n	%	n	%	n	%
H1 2021 (weeks 1–25)	1.	Beginning	21 634	0,50 %	18 037	0,60 %	3 597	0,20 %
		End	3 205 084	71,30 %	1 737 715	60,40 %	1 467 369	91,00 %
	2.	Beginning	0	0,00 %	0	0,00 %	0	0,00 %
		End	988 942	22,00 %	246 031	8,50 %	732 824	45,40 %
	3.	Beginning	0	0,00 %	0	0,00 %	0	0,00 %
		End	0	0,00 %	0	0,00 %	0	0,00 %
	4.	Beginning	0	0,00 %	0	0,00 %	0	0,00 %
		End	0	0,00 %	0	0,00 %	0	0,00 %
H2 2021 (weeks 26–52)	1.	Beginning	3 341 560	74,40 %	1 868 113	64,90 %	1 473 447	91,30 %
		End	4 022 243	89,50 %	2 486 928	86,40 %	1 535 315	95,20 %
	2.	Beginning	1 163 340	25,90 %	292 740	10,20 %	858 496	53,20 %
		End	3 859 371	85,90 %	2 348 025	81,60 %	1 465 419	90,80 %
	3.	Beginning	0	0,00 %	0	0,00 %	0	0,00 %
		End	1 278 162	28,50 %	461 347	16,00 %	806 245	50,00 %
	4.	Beginning	0	0,00 %	0	0,00 %	0	0,00 %
		End	902	0,00 %	372	0,00 %	522	0,00 %
H1 2022 (weeks 1–25)	1.	Beginning	4 029 078	89,30 %	2 492 890	86,60 %	1 536 188	94,00 %
		End	4 066 835	90,10 %	2 524 785	87,70 %	1 542 050	94,40 %
	2.	Beginning	3 875 746	85,90 %	2 359 474	82,00 %	1 471 087	90,00 %
		End	3 970 281	88,00 %	2 424 882	84,20 %	1 565 262	95,80 %
	3.	Beginning	1 632 259	36,20 %	707 818	24,60 %	904 734	55,40 %
		End	2 970 910	65,80 %	1 702 885	59,20 %	1 143 932	70,00 %
	4.	Beginning	2 428	0,10 %	1 236	0,00 %	1 160	0,10 %
		End	333 975	7,40 %	15 387	0,50 %	318 332	19,50 %
