# Supplementary material for: COVID-19 hospitalisations and all-cause mortality by risk group in Finland
Source: PLoS One. 2023 May 23;18(5):e0286142. doi: 10.1371/journal.pone.0286142 (PMC10204977; doi:10.1371/journal.pone.0286142)
Supplement: Attachment — Submitted filename: REVIEWERS COMMENTS-PLOS AB.docx [file pone.0286142.s008.docx]

**REVIEWERS COMMENTS- PONE-D-23-03744**

The study PONE-D-23-03744: Covid-19 hospitalisations and all-cause mortality by risk group” in Finland” aimed to identify which risk groups are at the highest risk of having
severe Covid-19 infection, sever disease leading to hospitalisation or death in Finland. The authors used retrospective observational study with Finnish registry data to answer this question.

On the whole, the manuscript is well written, clearly followed and easily understood.

Below are a few issues for authors consideration;

**Background**

Line 61: why Omicron in particular?

**Materials and methods**

Line 76-77: was the 2020 data unavailable especially the later months of the year

**Discussions**

Line 197-199: please cross-check this claim to be sure- there seems to many studies in that region reporting covid-19 severities and related mortalities vs risk factors

Line 201- 205:
