# Supplementary material for: COVID-19 hospitalisations and all-cause mortality by risk group in Finland
Source: PLoS One. 2023 May 23;18(5):e0286142. doi: 10.1371/journal.pone.0286142 (PMC10204977; doi:10.1371/journal.pone.0286142)
Supplement: Attachment — Submitted filename: PLOS ONE_Response to reviewers_rev.docx [file pone.0286142.s009.docx]

**Response to reviewers**

The authors’ comments can be found in red below.

5. Review Comments to the Author

Please use the space provided to explain your answers to the questions above. You may also include additional comments for the author, including concerns about dual publication, research ethics, or publication ethics. (Please upload your review as an attachment if it exceeds 20,000 characters)

Reviewer #1: First, I appreciate the authors efforts in conducting and presenting this interesting study. I understand that the peer review process can be challenging, but please rest assured that my aim is to provide constructive feedback that will help to improve the quality of your manuscript and contribute to its overall success.

Dear Reviewer,

Thank you very much for your thoughtful review of our manuscript. We greatly appreciate your kind words and your effort to provide constructive feedback that will help to improve the quality of our research. Your comments have been invaluable in shaping the final version of our manuscript, and we are grateful for the time and effort you have invested in this process.

1. Please use the term "COVID-19" instead of "Covid-19". This change has been made throughout the manuscript.
2. Abstract: The abstract presents a clear and concise overview of a retrospective observational study on COVID-19 in Finland, including the study's objectives, methods, and key findings. The abstract is written in a scientific style, and the language used is appropriate for a scientific audience. Overall, the abstract appears to be well-written and informative.
3. Introduction: Some minor improvements can be suggested as follows-
a) In the line 42, it should be noted that the World Health Organisation (WHO) announced COVID-19 as a global pandemic, not just "announced" it. This change has been made to the manuscript.
b) In the lines 44 and 45, it should be noted that while most people infected with COVID-19 have mild-to-moderate symptoms, a significant proportion may still experience severe symptoms leading to hospitalization and death. This change has been made to the manuscript.
c) In the line 46, it should be noted that while certain risk groups are more likely to develop severe COVID-19, the disease can affect anyone regardless of age or pre-existing conditions. This change has been made to the manuscript.
d) In the line 47, please add Chronic respiratory diseases as pre-existing condition, you may also add diabetes, because people with diabetes s and COVID-19 often need invasive ventilation care and need intensive care unit (ICU) due to their likelihood of developing Acute Respiratory Distress Syndrome (ARDS) -Aging (Albany NY). 2020 Apr 15; 12(7): 6049–6057. Published online 2020 Apr 8. doi: 10.18632/aging.103000) This change has been made to the manuscript and the suggested reference has been added.
e) In the line 53 and 54, it should be noted that while COVID-19 vaccines can reduce the burden of the disease, they do not completely eliminate the risk of severe symptoms or transmission. This change has been made to the manuscript.
f) In the line 56 and 57, it should be noted that while early treatment may prevent severe COVID-19, it is not always effective and should be used in combination with preventive measures and vaccination.
(Overall, the introduction provides a reasonable and accurate overview of the current understanding of COVID-19 and its risk factors, while acknowledging some uncertainties and the importance of ongoing research.) This change has been made to the manuscript.
4. Methods: There are no major scientific writing errors in the methods. However, here are a few minor suggestions for improvement-
a) In the line 72, the phrase "summary level statistics" could be clarified for readers who may not be familiar with the term. For example, "This study used aggregate data obtained from the Finnish Institute for Health and Welfare's registers..." This change has been made to the manuscript.
b) In the lines 83, 84 and 85, "The results are analysed based on patients identified from Hilmo..." could be rephrased for clarity, for example: "Data from Hilmo was used to identify patients for analysis, and any differences between TTR and Hilmo patients are reported in supplementary materials (S1 and S2 Tables)." This change has been made to the manuscript.
c) In the lines 113 and 114, it may be helpful to specify which types of visualizations were used for the descriptive data analysis. This change has been made to the manuscript.
d) In the line 119, it may be helpful to provide a brief explanation of the infection hospitalization rate (IHR) and the case fatality rate (CFR), for readers who may not be familiar with these terms. This change has been made to the manuscript.
e) In the lines 122 and 123, the phrase "divided by the total number of individuals with a COVID-19 infection" could be clarified by specifying whether this refers to the total number of infected individuals in the entire population or only within each risk group. This change has been made to the manuscript.
5. Results: Well written and described
6. Discussion:
a) In the lines 201 to 205, elderly with COVID-19 infection was only indentified as a risk group in this study? If not, please add some other risk factors points like Chronic lung diseases, diabetes, cardiovascular diseases. My suggestion stands: <https://doi.org/10.1371/journal.pone.0233147>; <https://doi.org/10.1016/S2213-2600(20)30116-8>. The other risk factors have been discussed now in the sentence earlier to this.

Reviewer #2: REVIEWERS COMMENTS- PONE-D-23-03744
The study PONE-D-23-03744: Covid-19 hospitalizations and all-cause mortality by risk group” in Finland” aimed to identify which risk groups are at the highest risk of having
severe Covid-19 infection, sever disease leading to hospitalization or death in Finland. The authors used retrospective observational study with Finnish registry data to answer this question.
On the whole, the manuscript is well written, clearly followed and easily understood.

Dear Reviewer,

We would like to express our sincere gratitude for your thoughtful review of our manuscript. Your kind words and feedback have been invaluable to us and we greatly appreciate your time and effort in providing us with your helpful comments. Thank you once again for your contribution to our manuscript. We have taken your feedback on board and made the necessary revisions to improve the quality of our work.

Below are a few issues for authors consideration;
Background
Line 61: why Omicron in particular? We wanted to focus on Omicron due to the fact that most of the published research has been about earlier virus variants. Additionally, the disease itself is different than during the earlier variants.
Materials and methods
Line 76-77: was the 2020 data unavailable especially the later months of the year The earlier data had been already reported and published in a research article by THL and therefore we wanted to focus on the newest datasets available (reference 8. Auro, K, Paajanen, T, Koskelainen, S, Vaara, S, Brunfeldt, M, Hannila-Handelberg, T et al. COVID-19-pandemian tunnusluvut Suomessa: ensimmäinen pandemiavuosi. Duodecim, 2022, 138.9: 821-830. Available from: <https://www.duodecimlehti.fi/xmedia/duo/duo16741.pdf>). Additionally, we wanted to include the time that vaccinations were already available in Finland: the vaccinations started early 2021 and the high-risk groups were prioritised in vaccinations.
Discussions
Line 197-199: please cross-check this claim to be sure- there seems to many studies in that region reporting covid-19 severities and related mortalities vs risk factors This has been cross-checked and to our knowledge only articles including data from 2020 have been published with some of the risk factors that we have also studied. Our study seems to be the first one from the Nordics reporting data from 2021 to 2022, including the omicron-dominant period of the pandemic.
Line 201- 205:
